# A fitness assay for comparing RNAi effects across multiple *C. elegans *genotypes

**DOI:** 10.1186/1471-2164-12-510

**Published:** 2011-10-17

**Authors:** Mark Elvin, Laurens B Snoek, Martin Frejno, Ulrike Klemstein, Jan E Kammenga, Gino B Poulin

**Affiliations:** 1 Faculty of Life Sciences, Michael Smith Building, The University of Manchester, Oxford Road, Manchester, M13 9PT, UK; 2 Laboratory of Nematology, Wageningen Universiteit, Droevendaalsesteeg 1, 6708 PB, Wageningen, The Netherlands

## Abstract

**Background:**

RNAi technology by feeding of *E. coli *containing dsRNA in *C. elegans *has significantly contributed to further our understanding of many different fields, including genetics, molecular biology, developmental biology and functional genomics. Most of this research has been carried out in a single genotype or genetic background. However, RNAi effects in one genotype do not reveal the allelic effects that segregate in natural populations and contribute to phenotypic variation.

**Results:**

Here we present a method that allows for rapidly comparing RNAi effects among diverse genotypes at an improved high throughput rate. It is based on assessing the fitness of a population of worms by measuring the rate at which *E. coli *is consumed. Critically, we demonstrate the analytical power of this method by QTL mapping the loss of RNAi sensitivity (in the germline) in a recombinant inbred population derived from a cross between Bristol and a natural isolate from Hawaii. Hawaii has lost RNAi sensitivity in the germline. We found that polymorphisms in *ppw-1 *contribute to this loss of RNAi sensitivity, but that other loci are also likely to be important.

**Conclusions:**

In summary, we have established a fast method that improves the throughput of RNAi in liquid, that generates quantitative data, that is easy to implement in most laboratories, and importantly that enables QTL mapping using RNAi.

## Background

**Figure 1:**
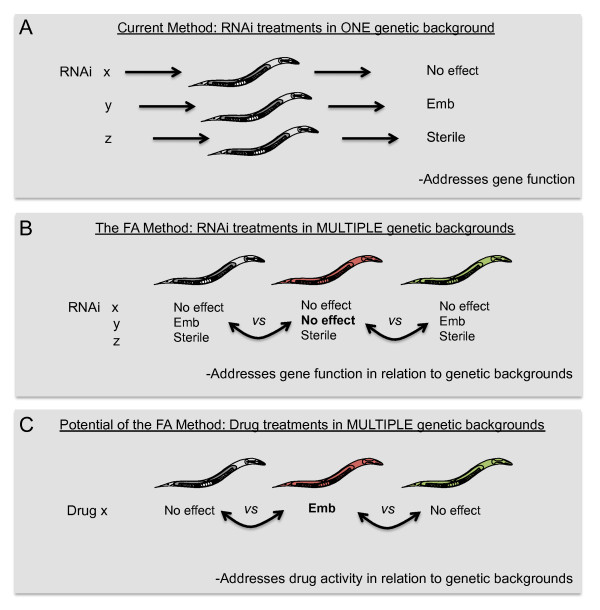
**The usefulness of the Fitness Assay**. A) RNAi by feeding in liquid has increased the throughput considerably to address gene function within a genetic background, but this method remains a qualitative or semi-quantitative method at best. B) The Fitness Assay is a method that measures the rate at which food is consumed. The Fitness Assay produces quantitative data from RNAi treatments for each genetic background tested. These data are then used to address gene function in relation to genetic backgrounds. C) Potentially, the Fitness Assay could be adapted to measure the effects of drug treatments on various genetic backgrounds and address drug activity in relation to these genetic backgrounds.

The first genome-wide RNAi screen performed in *C. elegans *proved to be a milestone, for the first time each predicted gene of a metazoan could be inactivated and the observed effects characterized in a systematic way [1]. Since then, steady improvements have been made to the RNAi feeding method, in particular the possibility to perform RNAi screens in liquid in a 96-well format [2]. The current methodology is appropriate to address gene function in either a wild type background or a mutated background. However, the current method becomes very difficult to apply when the question to be addressed is to determine the role of a particular gene in relation to multiple genetic backgrounds or genotypes (Figure 1). This problem mainly occurs because RNAi effects were not quantifiable when performed at a high throughput rate. We sought to solve this problem by developing a high throughput method that generates quantitative data fully amenable to statistical analyzes that allows us to tease apart the contribution of individual genetic backgrounds to a specific process.

The basic principle of the method is to assess the fitness of a population of animals by measuring the rate at which *E. coli *is consumed. Typically, an experiment assesses the fitness over a period of 8 days. This generates Fitness Curves (FCs), which are amenable to statistics. Various analyzes can be performed on the curves using parameters such as the slope, the mean, the time required to consume 50% of the food, the difference between first and last readings, or even the surface above the area of the curve. Here, we mainly used variation on the slope, since this parameter can capture most of the effects observed on the FCs.

We demonstrate that our approach is robust by mapping the *C. elegans *loss of RNAi sensitivity trait using diverse genetic backgrounds. We took advantage of Recombinant Inbred Lines (RILs) that were produced by mating two diverse genetic backgrounds N2 (Bristol) and CB4856 (Hawaii). This RIL population has extensively been used to map a range of quantitative traits [3-5]. Hence, we used these previously described RILs [6-8] and a number of new RILs (Additional file 1, Table S1). The N2 (Bristol) background is RNAi sensitive whereas the CB4856 (Hawaii) background is RNAi insensitive in the germline [9]. Using Quantitative Trait Loci (QTL) mapping, we identified *ppw-1*, a gene causal to the RNAi insensitivity of the CB4856 germline [9]. Importantly, we performed a detailed genotype-to-phenotype analysis that provides evidence that loss of RNAi sensitivity is a complex trait, in which *ppw-1 *is one of probably several RNAi sensitivity modifiers. In total, 56 genetic backgrounds were tested using 12 RNAi treatments. Analyzing such a number of genetic backgrounds has never been performed before, but critically this is the first time that quantitative data have been produced for RNAi treatments in *C. elegans*.

## Results

### Bristol (N2) and Hawaii (CB4856) respond differently to RNAi treatments

Bristol and Hawaii react very differently when exposed to RNAi. RNAi in Bristol is highly effective, but in Hawaii RNAi sensitivity for germline expressed genes has been lost [9]. Therefore, we reasoned that our fitness assay should be ideal to differentiate between these two RNAi sensitivity behaviors. For example, if we target an essential gene that functions in the germline, Bristol will die, but not Hawaii. We performed RNAi in both strains on a panel of 12 RNAi clones: four clones (targeting *par-1*, *par-6*, *pos-1*, and *mel-26*) are known to be more effective in Bristol than in Hawaii [9]; two clones (targeting *rab-5 *and *tag-214*) are known to be effective in both natural isolates [9]; one clone (targeting *lin-31*) is used as a negative control since it affects vulval development, a non-essential organ for viability [10]; four clones (targeting *smo-1*, *mpk-1*, *gld-1*, and *let-502*) have never been tested before for comparison between Bristol and Hawaii; and the empty vector clone is used as a control or a reference. We have analyzed the data generated from the fitness assay in two ways: i) by comparing each RNAi condition to the empty vector for each strain and ii) by analyzing whether Bristol responds differently from Hawaii to specific RNAi treatments.

**Figure 2:**
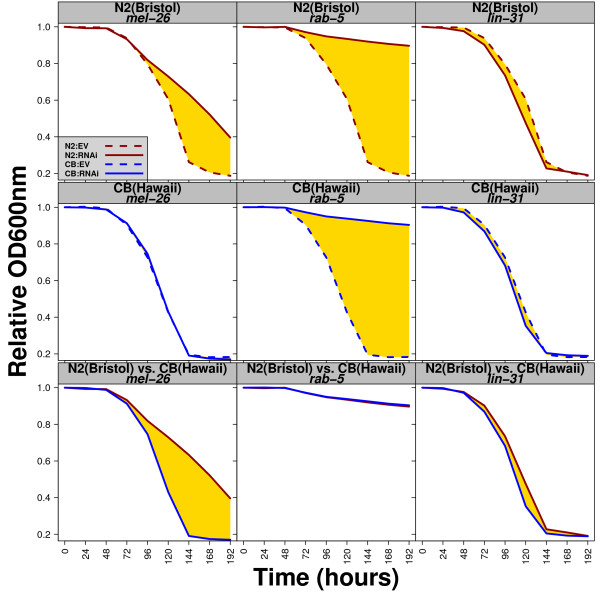
**Comparison of RNAi treatments between Bristol and Hawaii**. Shown are three representative RNAi treatments that illustrate RNAi effects in Bristol (upper panel), in Hawaii (middle panel), and their differential effects in Bristol *versus *Hawaii (lower panel). The negative control is *lin-31(RNAi) *and the reference is the empty vector (ev). Relative OD at 600 nm on the y-axis, time (in hours) on the x-axis. The RNAi treatment is indicated in the grey box. N2 is shown in red, CB in blue.

In the first part of the analysis, we found that eight out of eleven RNAi treatments caused a reduction in fitness in Bristol (Figure 2 (top panels show three examples) and Table 1). These RNAi treatments have been previously shown to cause lethality, to reduce fertility, or to reduce growth rate [1], all these parameters are predicted to affect the rate of food consumption. We were not able to detect an effect with *let-502 *and *mpk-1(RNAi)*, indicating that RNAi conditions in liquid may have been less efficient for these specific cases. RNAi by feeding has been shown to produce a high rate of false negative results but a very low rate of false positive results [1]. We next analyzed the FCs produced in the Hawaii strain. Only the *rab-5(RNAi) *and *tag-214(RNAi) *treatments can produce FCs that are significantly different from the reference (ev), likely because these genes are functional outside the germline (Figure 2 (middle 3 panels) and Table 1).

**Table 1:** Three-way analysis of RNAi treatments performed on Bristol (N2) and Hawaii (CB4856)

gene targeted	EV vs Target in Bristol	EV vs Target in Hawaii	P value (Bristol vs Hawaii)
**ev**	-	-	0.15
*mpk-1*	0.80	0.86	0.10
*smo-1*	**9.80E-06**	0.74	**5.30E-08**
*par-1*	**0.04 **^**1**^	0.74	**0.0013**
*lin-31*	0.50	0.35	0.097
*let-502*	0.09	0.50	**0.03**
*gld-1*	**0.039**	0.79	**1.30E-06**
*par-6*	**0.022**	0.36	**0.0025**
*pos-1*	**0.007 **^**1**^	0.67	**9.20E-06**
*rab-5*	**6.10E-33**	**4.50E-36**	0.23
*mel-26*	**2.18E-09**	0.93	**1.70E-14**
*tag-214*	**2.56E-16**	**4.90E-29**	**8.90E-15**

The second column indicates differences that can be detected between the RNAi treatment and the control (ev) in Bristol. P values are derived from time points 5 to 9 except for ^1^, in which time points 6 to 8 were used (see summary statistics). The third column indicates differences that can be detected between the RNAi treatment and the control (ev) in Hawaii. The last column is a comparison between Bristol and Hawaii.

In the second and most innovative part of our analysis, we compared the FCs obtained from the same RNAi treatment between Bristol and Hawaii. Accordingly, Bristol and Hawaii do not respond differentially to empty vector, *mpk-1(RNAi)*, the control *lin-31(RNAi)*, and *rab-5(RNAi) *(Table 1). However, we observed differential responses with RNAi targeting *mel-26*, *pos-1*, *par-1*, *par-6*, *gld-1*, *smo-1 *and *let-502 *(Figure 2 (bottom 3 panels) and Table 1). Surprisingly, *tag-214 *appears to produce a differential response suggesting that the RNAi treatment was more effective in Hawaii than Bristol. Detailed analysis would be required to confirm this observation. Taken together, these results show that RNAi treatment effects can produce data consistent with previous studies [1,2]. Importantly, these data can be quantified when applied onto diverse genetic backgrounds or genotypes.

### PPW-1 is a modifier of RNAi sensitivity

**Figure 3:**
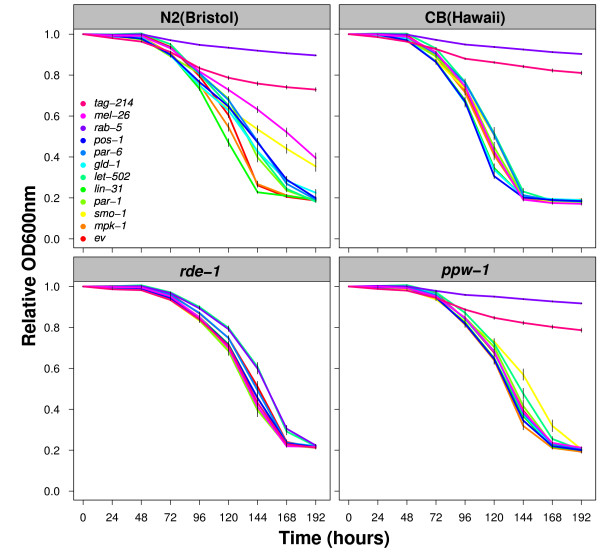
**The average FCs for Bristol, Hawaii, *rde-1 *and *ppw-1 *mutants following empty vector (ev) and eleven RNAi treatments**. N2 is affected by many different RNAi treatments. *rde-1 *is not affected by any RNAi treatment. CB and *ppw-1 *are affected by the same RNAi treatments. Strains are indicated in the grey boxes on top of each panel. RNAi treatments are indicated by the different colors, legend in left upper panel. Time in hours on the x-axis and the relative OD at 600 nm on the y-axis.

The differential effect for germline RNAi sensitivity observed between Bristol and Hawaii is attributed to PPW-1 [9]. PPW-1 is an RNA binding protein of the PIWI family, which is important to maintain RNAi sensitivity of genes presumably active in the germline. However, RNAi sensitivity can also be mediated at a general level, such as in the case of an *rde-1 *mutant, in which RNAi sensitivity is totally lost [11,12]. To distinguish between these general and germline-specific effects, we tested *rde-1 *and *ppw-1 *mutants and compared them with Bristol and Hawaii, respectively. Looking at the FCs, we can readily detect that the *rde-1 *mutant lost total RNAi sensitivity (Figure 3). Therefore, all the RNAi effects observed in Bristol require the general RNAi machinery and cannot be attributed to generic culture condition problems. In contrast, the *ppw-1 *mutant FCs nicely phenocopies the FCs obtained from the Hawaii strain (Figure 3). Therefore, loss of RNAi sensitivity in Hawaii is consistent with *ppw-1 *deleterious polymorphisms [9].

### RNAi treatments of 56 RILs identifies a shared QTL at the *ppw-1 *locus

**Figure 4:**
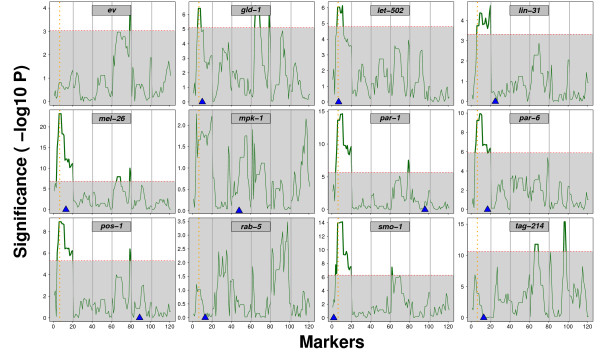
**A shared QTL is found close to *ppw-1***. The RNAi treatment is indicated in the grey box at the top of each individual panel. The significance (y-axis) of the marker explaining the variation in the FCs is plotted against the marker position (x-axis). Significance is shown as -log10 of the p-value per marker. Chromosome borders are indicated by the grey vertical lines. The positions of the RNAi targeted genes are indicated by the blue triangle on the x-axis. The vertical orange dotted line indicates the position of *ppw-1*, a gene polymorphic between N2 and CB, known to be involved in germline RNAi sensitivity [9]. The genome-wide threshold (0.05 after 1000 permutations) is indicated by the horizontal dashed red line. The part of the QTL profile below the threshold was plotted on a grey background. For both the QTL calculations and threshold determination see methods. Critically, a shared QTL close to the *ppw-1 *locus is observed between *mel-26(RNAi)*, *pos-1(RNAi)*, *par-1(RNAi)*, *smo-1(RNAi)*, *par-6(RNAi)*, *gld-1(RNAi) *and *let-502(RNAi)*.

Polymorphisms in *ppw-1 *appear to explain the loss of germline RNAi sensitivity in Hawaii. However, even though the *ppw-1 *mutant behavior is consistent with this view, the previous study [9] did not address or rule out whether other natural modifiers exist. Since our method is able to detect differential RNAi behaviors at a quantitative level, we tested if we could map the loss of germline RNAi sensitivity trait using QTL analysis. For this purpose, we exposed 56 RILs generated from a cross between Bristol and Hawaii [6-8], and their respective parental strains, to our 12 RNAi treatments. Interestingly, we found a highly significant QTL on Chromosome *I*, close to the *ppw-1 *locus (Figure 4). This QTL is especially strong for *mel-26(RNAi)*, *smo-1(RNAi)*, *par-1(RNAi)*, *par-6(RNAi) *and *pos-1(RNAi) *and harbours *ppw-1*.

### A genotype-to-phenotype analysis suggest that loss of RNAi sensitivity is a complex trait

**Figure 5:**
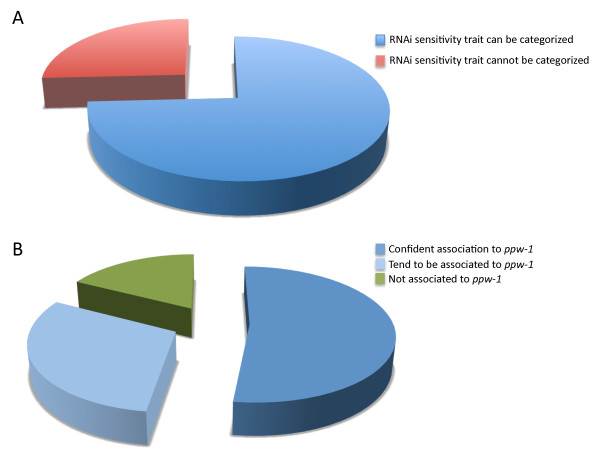
**RNAi sensitivity is a complex trait mostly associated to *ppw-1 *polymorphisms**. A) We genotyped 31 RILs at the *ppw-1 *locus and addressed whether each of these RILs are RNAi sensitive or not using FCs from *mel-26(RNAi), pos-1(RNAi), par-1(RNAi), smo-1(RNAi), par-6(RNAi)*, and *gld-1(RNAi)*. This analysis has revealed that 25.8% of RILs cannot be categorized as such, underlying that the RNAi sensitive trait is complex. However, 74.2% of these could be categorized as either N2-like or CB-like. B) From the RILs that can be categorized, most cases (82,6%), can be associated to the correct *ppw-1 *allele. On the other hand, only 17.4% of the cases are not significantly associated with *ppw-1*. Therefore, *ppw-1 *is a strong predictor of RNAi sensitivity, but not the only one.

Even though *ppw-1 *was previously identified as an important contributor to loss of RNAi sensitivity, and that our results so far support this view, we tested at an individual level how well correlated the *ppw-1 *genotype is to the loss of germline RNAi sensitivity phenotype. We first sequenced the *ppw-1 *locus for 31 RILs and then analyzed whether the genotype can predict the phenotype, herein loss of germline RNAi sensitivity. The prediction is that a genotype that matches with Hawaii *ppw-1 *polymorphisms should cause loss of RNAi sensitivity in the germline. *Vice versa*, the RILs with Bristol *ppw-1 *polymorphisms should remain RNAi sensitive. Surprisingly, we found that in about 25% of the cases, we could not categorize the phenotype to either Bristol-like or Hawaii-like behavior (Figure 5A). However, if we focused on the 23 RILs (~75%) that can be categorized as either Bristol-like (12 RILs) or Hawaii-like (11 RILs), we found that in ~83% of the cases, the loss of RNAi sensitivity trait correlates to Hawaii *ppw-1 *(Figure 5B). Taken together, these results provide new evidence that loss of RNAi sensitivity in Hawaii is not entirely due to polymorphisms in *ppw-1 *and that other important RNAi sensitivity modifiers must exist, even though *ppw-1 *is strong predictor of germline RNAi sensitivity [9].

### Factors affecting the fitness assay

Here we show that our method is efficient at addressing the contribution of genetic variation on a specific trait, herein loss of germline RNAi sensitivity. We think that the Fitness Assay will be useful to other groups interested in identifying the contribution that diverse genotypes may have to their phenotypes of interest. This may involve different treatments such as environmental stress or chemical compounds. The Fitness Assay is easy to implement in any *C. elegans *laboratory, but future users need to take into account two critical parameters for future adaptations of the fitness assay: the number of worms and the quantity of food. These parameters need to be optimized, in particular for strains displaying a low brood size or lethality.

For our purpose, the worms were seeded manually, but a multiwell dispenser could also be used. This apparatus can process a 96-well plate in about 10 seconds, but tends to be more variable than the manual method using a single channel pipette (+/- 4.5 versus +/- 3 worms, respectively). We aimed at seeding 20 L1 worms per well for our experiments, and used duplicates for the manual method, however when using the multiwell dispenser we performed the experiments in triplicate. We directly tested the efficiency of our assay using the multiwell dispenser by performing six RNAi treatments (*lin-31, rab-5, smo-1, par-1, par-6, and mel-26*) over 38 RILs. We found that we can still map a QTL at the *ppw-1 *locus for *smo-1, par-1, par-6 and mel-26 *RNAi treatments (Additional file 2, Figure S1). This confirms that the multiwell dispenser can also be used to seed the worms to perform the Fitness Assay. Even though not tested here, it is very likely that using a worm sorter, such as the COPAS (Union Biometrica), could reduce or perhaps eliminate the worm number issue and increase resolution.

**Figure 6:**
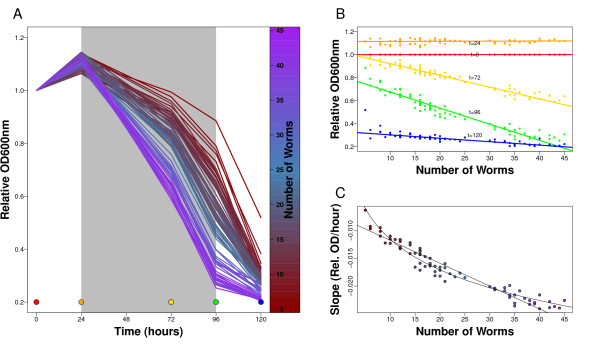
**Effect of number of worms seeded on the Fitness Assay**. A) Color of the FCs represent the number of worms. Colored dots represent the time-points. Grey area shows the time where the slopes of the curves are most distinctive for the number of worms. B) The slope of the linear relation between worm-number and relative OD is the steepest at 72 and 96 hours. Therefore the time frame between 24 and 96 hours is most distinctive for the number of worms when the slope of the curves is used. C) Number of worms plotted against slope of the relative OD between 24 and 96 hours. The variation in decrease of OD per hour is between approximately -0.025 and -0.0175 when 20 +/- 5 are taken. The maximum possible variation by different worm number is +/- 0.0025 OD per hour. From the linear relation of the slope and the number of worms one can calculate 'a comparable number of worms'. We explained the slope by the number of worms with a linear model. The standard deviation of the effect of the number of worms on the slope divided by the mean effect of the number of worms on the slope give a factor that can be used to determine a comparable number of worms. For the linear model this factor was 0.29. For example if 1sd deviation from the mean slope is accepted for a mean of 20 worms any number between 20 -/+ 5.8 can be used.

The number of worms is an important factor in the Fitness Assay, but to what extent can it affect the interpretation of the data? To answer this point, we next addressed the sensitivity limit of the Fitness Assay by assessing the effect of worm numbers on the Fitness Curves (Figure 6A and 6B). We have found that if the number of worms seeded has a variation of +/- 5 and that the average number of worms is 20, the variation produced on the slopes is +/- 0.0025 OD/hours (Figure 6C). Hence, changes within that range must be repeated/checked since the effect may be due to the variation on the number of worms seeded. On the other hand, an effect on the slope beyond this variation is very likely to be attributed to the applied treatment, herein RNAi. In the experiment using the multiwell dispenser, the effects observed for RNAi treatments that show a QTL on *ppw-1 *are far beyond the variation caused by worm number variation. For example the four genes *smo-1*, *par-1*, *par-6*, *mel-26*, with a QTL on *ppw-1 *show an allelic difference for this parameter between the Hawaii and Bristol of respectively 0.0043, 0.0051, 0.0036, 0.0048 OD/hours, which is about two fold above the maximum variation possibly caused by varying worm numbers. We are therefore confident that the calculated effects on the Fitness Assay are caused by RNAi treatments.

**Figure 7:**
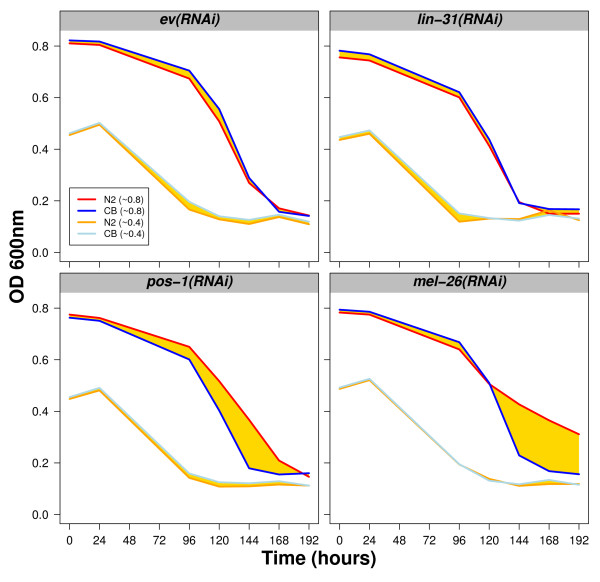
**Loss of RNAi effects if the initial concentration of food is too low**. Bristol or Hawaii worms were RNAi-treated with empty vector (ev)*, lin-31, pos-1 *or *mel-26*, as indicated. Both *pos-1(RNAi) *and *mel-26(RNAi) *effects were lost if about half the initial concentration of food was used.

Another important parameter is the quantity of food. We determined that for RNAi a starting OD of around 0.8 units is appropriate. We have also found that if RNAi is performed with half that amount of food, RNAi effects are affected (Figure 7). Indeed, the differential effect detected between Hawaii and Bristol disappears when the worms are treated with *pos-1(RNAi) *or *mel-26(RNAi) *(Figure 7). In summary, the ratio worms versus food is important and it may need to be adjusted according to different strains and food sources used.

### Sources of variation

**Figure 8:**
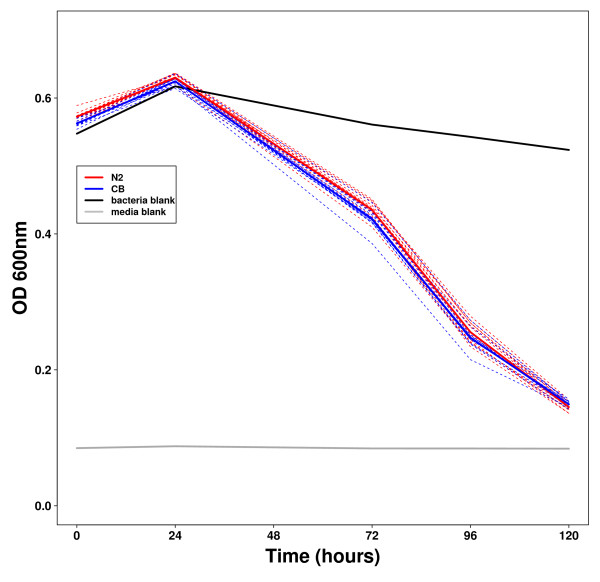
**Technical reproducibility of the Fitness Assay using Bristol and Hawaii**. Both strains were seeded in separate 96-well plates and curves were derived for each well. Bacteria blanks are wells without worms, and media blanks are wells without worms and bacteria. This shows that bacteria do not grow or die significantly without worms and that the worms' absorbance at 600 nm is slight.

As with any technique there is an associated technical variation. We addressed the extent of this by repeating, within a same experiment, multiple times the Fitness Assay. We found this technical variation to be minimal (Figure 8). However, there is a degree of variation between experiments (batches) but this is easily accounted for by systematically adding the reference or parental strains. We address this point in the materials and methods section. To sum up, it is critical that each Fitness Assay experiment is self-contained, *i.e*. includes positive and negative controls for the specific treatment, herein RNAi, and includes the reference strains, herein Bristol and Hawaii.

## Discussion

Here we have presented a new assay that is both cost efficient and easy to set-up in any laboratory. The main advantage of the Fitness Assay is its ability to distinguish and quantify the effect of a specific treatment across multiple genetic backgrounds or genotypes (Figure 1). We show that this is particularly powerful to identify the QTL responsible for loss of germline RNAi sensitivity in RILs generated from Bristol (RNAi sensitive) and Hawaii (RNAi insensitive) [6-9]. This study provides evidence that modifiers of this trait remain to be identified. The method is also versatile and other treatments could be used to decipher the contribution of natural genetic variation to specific traits, *i.e. *a drug response.

The Fitness Assay indicates the capacity of an animal population to eat at a certain rate. This rate is determined by multiple factors, including the number of progeny, the level of viability, and the rate of growth. In our selected set of RNAi, we found that most targeted genes produced a significant effect on the FCs (Fitness Curves), which is in accordance with their previously described function. In addition, using data from Kamath et al, 2003, we found that most phenotypes, around 75%, are due to embryonic lethality, a reduced brood size, or a growth defect. Indicating that the majority of genes that produce a phenotype by RNAi in Bristol could be identified by our method. We tested this prediction by targeting 40 genes: 20 known to produce 'viability-related' defects, and 20 with no 'viability-related' defects reported by [1]. However, these latter 20 targets have been shown to have functions associated with signaling pathways, apoptosis or transcription (see Wormbase). We found that 75% of the genes required for viability produced a phenotype in the Fitness Assay, indicating that most RNAi treatments that produce a 'viability-related' phenotype would also produce a phenotype in the Fitness Assay (Additional file 3, Figure S2). Using the other set of targeted genes, we found that 60% of the RNAi treatments have no effect. Interestingly, the remaining 40% producing an effect on the Fitness Assay have been shown to play roles in WNT signaling (*lin-44*), nuclear excision repair (*xpg-1*), Notch signaling (*sup-17 *and *aph-1*), apoptosis (*ikb-1 *and *vps-18*), translational repression (*fbf-1*), and transcription (*mab-5*) (Additional file 4, Figure S3). Collectively, these data indicate that the phenotypes identified using the Fitness Assay mostly overlap with the data from Kamath et al. 2003 [1] and that overall if we were to re-perform a genome-wide screen, we would miss some, but also detect a few new ones.

Even though phenotypes confined to non-viable organs such as the vulva could be missed by our method, the signaling pathways involved are often functional in other organs, and these organs may be required to maintain viability. For example, vulval development requires the RAS signaling pathway, but this pathway is also essential to the development of the excretory cell; and a malfunction of the excretory cell will cause larval lethality [13]. The Fitness Assay can identify this phenotype. There are alternatives methods that provide high-resolution phenotypic analysis. For example, high throughput image analysis increases phenotypic information, albeit at a reduced throughput [14,15]. However, most *C. elegans *laboratories do not have an up and running automated image-capture system. For these laboratories, the Fitness Assay remains the affordable option.

## Conclusions

In summary, this is the first study that shows that RNAi effects can be quantified in *C. elegans*. This is important since it will allow us to understand the contribution of diverse genetic backgrounds to complex traits. For example, there is the concept that medicine should be personalized, in part because of genetic variation between humans. Perhaps fundamental principles of genetic variation could be derived from studies performed in Recombinant Inbred Lines in *C. elegans*, and these could help develop the field of personalized medicine research.

## Methods

### Experimental set-up and timeline

**Figure 9:**
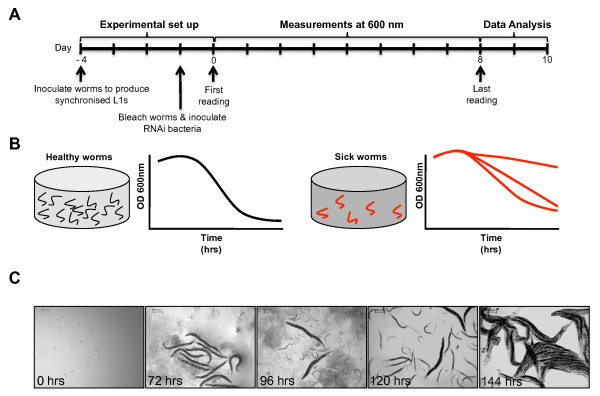
**Schematic representation of the Fitness Assay**. A) Timeline of the Fitness Assay. About two weeks should be allowed from the inoculation of worms from which synchronized L1s will derive to the complete analysis of the data. B) Depiction of the Fitness Curves (FCs) produced by wild type worms (left) or sick worms (right). Different FCs from sick worms have been observed. This seems to depend on the penetrance and the type of phenotypes, e.g. low brood sizes, slow growth, embryonic lethality or sterility. C) Pictures of the N2 worms seeded for a typical Fitness Assay taken at 0, 72, 96, 120, and 144 hours showing that the experiment starts with synchronized L1s, that these will produce progeny visible at 72 hours, and that these progeny are growing. For each picture, the worms are taken out of a well (from a 96-well plate) and seeded on an NGM plate without food to reduce bacterial interference during photography.

The Fitness Assay takes two weeks from inoculation of the worms to the statistical analysis of the FCs (Fitness Curves) generated (Figure 9). Briefly, we used L1 worms seeded at about 20 worms per well and performed the experiment in duplicate. It is critical to avoid bacterial or fungal infection since these will affect the readings. We always read the optical density (OD) at the start of the experiment (Day 5) and the following days as indicated in Figure 9A. We also found that the food consumption rate is very low between day 7 and day 8, therefore readings at these time points can be omitted without affecting the analysis. At days 11 and 12, we performed a visual inspection under the dissecting microscope to ensure that the experiment is of a good standard, *i.e. *shows no sign of contamination or infection. If this happens, normally most plates and wells are affected. At the end, healthy worms produce a characteristic FC (Figure 9B, left panel). Worms subjected to RNAi treatments causing sterility, embryonic lethality, low brood size or slow growing phenotypes will produce FCs that lose this characteristic shape (Figure 9B, right panel). Hence the Fitness Assay principle is that normal worms will reproduce and consume their food at a different rate (i.e. faster) than worms displaying phenotypes that affect viability, fertility and growth rate.

### Strains and growth conditions

Wild type *C. elegans *strains (N2 Bristol and CB4856 Hawaii) were used. Other strains: NL2550 *ppw-1(2505) I *and WM27 *rde-1(ne219) V*. For QTL mapping we used a total of 56 Recombinant Inbred Lines (RILs) generated from a cross between Bristol and Hawaii, of which 27 are partly described in [6-8]. We added 29 newly generated RILs, the genotype of each RIL used is described in Additional file 1, Table S1. All strains were maintained on *E. coli *OP50-seeded Nematode Growth Medium (NGM) plates as previously described [16]. All experiments were conducted at 20°C.

### RNAi by feeding in liquid 96-well format

#### 1. Preparation and induction of RNAi bacteria

Inoculate RNAi bacteria into 1 ml of LB containing 100 μg/ml ampicillin in a deep 96-well plate (BD Biosciences 353966) and incubate overnight at 37°C in a shaking incubator at 250 rpm. To induce the production of dsRNA add IPTG to a final concentration of 4 mM and incubate at 37°C for 1 hr in a shaking incubator at 250 rpm. After 1 hr pellet the bacteria by centrifugation at 4000 rpm for 5 mins. Resuspend bacterial pellets in 200 μl S-medium containing 100 μg/ml ampicillin and 4 mM IPTG [2].

#### 2. Preparation of worm strains

On the day prior to setting up RNAi by feeding in liquid bleach gravid adults and hatch embryos overnight in M9 buffer to obtain synchronized L1 population. The following day pellet synchronized L1 worms by centrifugation at 4000 rpm for 1.5 mins and resuspend in M9 for a concentration of approximately 20 L1 worms per 5 μl of M9 buffer.

#### 3. RNAi by feeding in liquid

For direct comparisons to be made between strains under study then approximately 20 synchronized L1 worms should be added to each well of a 96-well plate (Corning^® ^Costar^® ^CLS3596) in 5 μl of M9 buffer (either with a pipette or with the multiwell dispenser). Immediately afterwards add 60 μl of the resuspended RNAi bacteria in S-medium (from 200 μl volume). Perform all RNAi experiments in triplicate (therefore using 180 μl out of the 200 μl bacterial resuspension). Incubate 96-well plates in a humidity chamber at 20°C for the duration of the experiment.

#### 4. OD measurements using plate reader

Measure the absorbance of the triplicate 96-well plates at 600 nm using a plate reader (Biotek^® ^EL808) between 0 hrs - 192 hrs (0 - 8 days).

### Normalization of FC data (with unequal start ODs)

Within an RNAi treatment the sources of variation could be either technical or genotypic. When testing RILs our interest is in the genotypic variation, therefore it is desirable to correct for some of the technical variation. Each of our experiments was performed in triplicate, always including Hawaii and Bristol as reference strains, and additionally included a row dedicated to RNAi bacteria without worms (bacteria blank). Hence, controlling for contamination and spurious bacterial growth, respectively. We observed that variation of bacterial growth (without worms) is minimal between different replicas, experiments or treatments. However, the starting ODs can vary (mean: 0.79 sd: 0.065). Therefore, we investigated if the bacterial growth was affected by the start OD. The 312 growth curves of all the batches of the RNAi bacteria without the worms were taken together and the source of variation studied by an anova. The linear model used was: " y(OD) ~ x(time) + x(time):x(start OD) ". This showed that, although the starting OD is a highly significant (p < 1*10^-16^) source of variation in the bacterial growth curves the effect size is minimal. The growth curves are nearly parallel and indicate no significant bacterial growth. However, to correct for the variation observed between starting OD, we divided the raw ODs of all time-points by the OD of the first time-point. In this way all curves start at relative OD of 1.

The start OD of the bacterial suspensions in which the worms were exposed to RNAi were also different (mean: 0.83; sd: 0.069). Above we have shown that this could influence the FCs. To normalize and correct for this difference, we compared the significance and the effect of the start OD on the variation found in the uncorrected and the start OD corrected FCs. All the 160 empty vector FCs were taken together and used in an anova. The linear model used was: " y(OD) ~ x(time) + x(time):x(start OD)". Both the significance and the effect of the start OD interaction with the slope of the FCs were lower when the FCs were corrected by dividing by the start OD (sig: 1.8*10^-28 ^→ 5.6*10^-8 ^; eff: 5.4*10^-3 ^→ 3.0*10^-3^). A similar reduction of the role of the start OD was found after correction for specific RNAi used (data not shown).

We further investigated the effect of the correction of the ODs on genotype as an explanatory factor for the variation in the feeding curves. All the RNAi data was analyzed together in an anova. The model used in the anova was: " y(OD) ~ x(time) + x(genotype) + x(RNAi) + x(time):x(start OD)". The start OD is far less significant after correction whereas the genotype as source of the variation is as significant as when the uncorrected values are used. We conclude that it is best to start with equal amounts of food (start OD) but since this is not always possible, it is beneficial to correct for the start OD when analyzing the feeding rate dynamics.

### Differences between the FC signatures of Empty Vector and RNAi Treatment and between the FC signatures of Bristol and Hawaii

We calculated the significance of the difference between the FC curves of empty vector (ev) and RNAi treatments or between N2 and CB by a chi-square test. The individual replicates of the samples under comparison are taken together, normalized and the mean relative OD per time point is calculated. Then for each replicate it is scored per time point for whether the relative OD is above or below the mean. The sum of scores is taken and (as an example) a matrix is built (Table 2). This matrix is used to perform a chi-square test. For example, if samples 1 and 2 have similar values, the chi-square test will indicate that they are not statistically different. On the other hand, if samples 1 and 2 have different values, the test will indicate that they are statistically different.

**Table 2:** Matrix used to perform a chi-square test

	< mean	> mean
sample 1	12	24
sample 2	30	6

The (< mean) indicates the number of replicates below the mean and the (> mean) indicates the number of replicates above the mean.

### Genotyping the new recombinant inbred lines

The new recombinant inbred lines were genotyped for 96 SNPs by Illumina "Golden gate" SNP genotyping [17] (Additional file 1, Table S1). SNPs correspond to previously used SNPs to genotype 80 RILs [6]. Information from Illumina can be found at: (http://www.illumina.com/technology/goldengate_genotyping_assay.ilmn)

### QTL mapping

All FC data were normalized per RNAi treatment. ODs per time point of all experiments were divided by the average start OD. In this way all RNAi treatments had a relative start OD of 1 in all replicate experiments. We calculated the significance of each of the 121 markers of the N2/CB RIL population (partly described in [6-8]) by a linear model for each of the individual RNAi treatments and the empty vector (ev). With this linear model the QTLs are calculated by explaining the variation in FCs by start OD, time and marker (y(OD)) ~ x(time) + x(time):x(startOD) + x(marker). All measured FCs were used and only the approximate linear part of the FC was used (time-points: 24 to 196). We used 1000 permutation per RNAi treatment to determine a genome-wide -log10(p) threshold of 0.05. The FCs were randomized over the RILs for each round of QTL mapping in the permutation test. The QTL profiles were collected and for each profile the most significant score was put in a list. This list was ordered and the 50^th ^highest value per RNAi treatment was used as the threshold.

QTL mapping for the 6 RNAi treatments using the multiwell dispenser (Additional file 2, Figure S1) was done by calculating the slopes of the FCs over time-points 2 to 5 (24, 96, 120 and 144 hours) of the three replicas and than take the average per genotype. We used 38 RILs to calculate the QTLs with a single marker model: y(mean slope)~x(marker).

### Comparing FCs of individual RILs to Bristol and Hawaii

The FCs of 32 individual RILs, for which we sequenced *ppw-1*, were compared to the FCs of Bristol and Hawaii by a chi-square test. Averages per strain per time-point were calculated and normalized by dividing by the start OD. The FCs were further transformed to % OD of the normalized start OD. Two separate tests were performed for the RILs FCs against the Bristol's FCs and Hawaii's FCs.

### Statistical summary

As with any high-throughput experiment, the Fitness Assay generates large quantities of data. The nature of this data enables many types of detailed analysis. But to be used as a truly high-throughput method, a simple robust type of analysis needs to be applied. Also a software environment that enables high-throughput of statistical tests is recommended. The simplest level of analysis is to reveal the most severe RNAi effects. To this end, we used a t-test, which is performed on the relative ODs of the last time point(s). The next level of analysis aims at detecting less severe RNAi effects, which tend to affect the middle part of the curves. We found that comparing slopes using a t-test reflects the RNAi treatment effects better. Alternatively, a chi-square test can be used on the mean curve of the two samples. The time points of the individual replicates are tested to be above or below the overall mean and summed per treatment. The advantage of this latter method is that it can be used regardless of the shape of the curve and that it is easily adjusted to a specific part of the curve. In summary, many different tests can be applied to analyze the FCs, but in most cases simple tests are sufficient to detect RNAi treatments or genotypes effects.

## Authors' contributions

ME and UK performed all the RNAi experiments and strain characterizations. ME analyzed the data and supervised UK. MF initiated the development of the Fitness Assay. GP conceptualized the Fitness Assay, supervised and aided in data analysis. LBS analyzed the data and performed the QTL mapping and JK supervised and aided in the analysis. GP, JK, ME and LBS wrote the manuscript. All authors read and approved the final manuscript.

## Supplementary Material

Additional file 1 **Newly generated recombinant inbred lines between Bristol and Hawaii**. The new RILs have been genotyped using the indicated markers **Table S1**. Newly generated recombinant inbred lines between Bristol and Hawaii Click here for file

Additional file 2 **QTL profiles of six RNAi treatments performed using a multiwell dispenser**. QTL mapping of *ppw-1 *using 38 RILs **Figure S1**. QTL profiles of six RNAi treatments performed using a multiwell dispenser. QTLs were calculated on the slope of the FCs. The slopes were derived using time-points 2 to 5 and averaged per RIL. Thresholds (0.05) were determined by 300 permutations per treatment and are shown as a red horizontal line. The physical position of *ppw-1 *is indicated by a green dot. The RNAi treatment is indicated in the grey box. Click here for file

Additional file 3 **20 randomly selected RNAi treatments predicted to affect viability**. Fitness Curves of RNAi treatments **Figure S2**. 20 randomly selected RNAi treatments predicted to affect viability by RNAi according to [1]. Shown are the FCs of RNAi treatments (blue line) compared to empty vector (grey line). The difference between the RNAi effect and the control empty vector (ev) is shown in yellow. The RNAi treatment on N2 is indicated in the grey box. Individual measurements are shown as dots, blue for RNAi treatments and grey for ev. Click here for file

Additional file 4 **20 randomly selected RNAi treatments predicted to NOT affect viability**. Fitness Curves of RNAi treatments **Figure S3**. 20 randomly selected RNAi treatments predicted to NOT affect viability by RNAi according to [1]. Shown are the FCs of RNAi treatments (blue line) compared to empty vector (grey line). The difference between the RNAi effect and the control empty vector (ev) is shown in yellow. The RNAi treatment on N2 is indicated in the grey box. Individual measurements are shown as dots, blue for RNAi treatments and grey for ev. Click here for file
