# Supplementary figures and images for: A fitness assay for comparing RNAi effects across multiple C. elegans genotypes
Source: BMC Genomics. 2011 Oct 17;12:510. doi: 10.1186/1471-2164-12-510 (PMC3206879; doi:10.1186/1471-2164-12-510)

Signif. ( $-\log_{10}(p)$ )

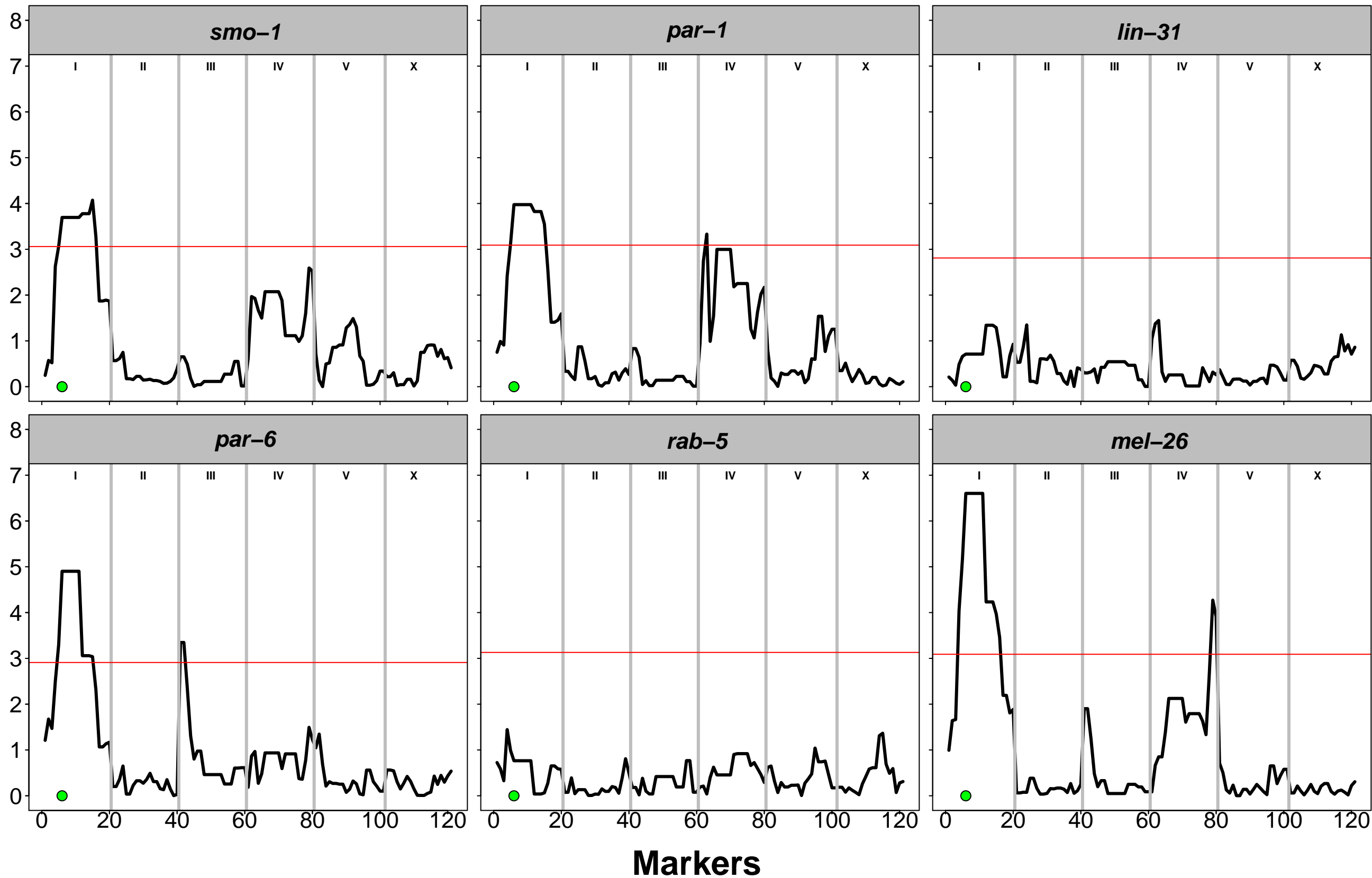

Supplement: Additional file 2 — QTL profiles of six RNAi treatments performed using a multiwell dispenser. QTL mapping of ppw-1 using 38 RILs Figure S1. QTL profiles of six RNAi treatments performed using a multiwell dispenser. QTLs were calculated on the slope of the FCs. The slopes were derived using time-points 2 to 5 and averaged per RIL. Thresholds (0.05) were determined by 300 permutations per treatment and are shown as a red horizontal line. The physical position of ppw-1 is indicated by a green dot. The RNAi treatment is indicated in the grey box. [file 1471-2164-12-510-S2.PDF]

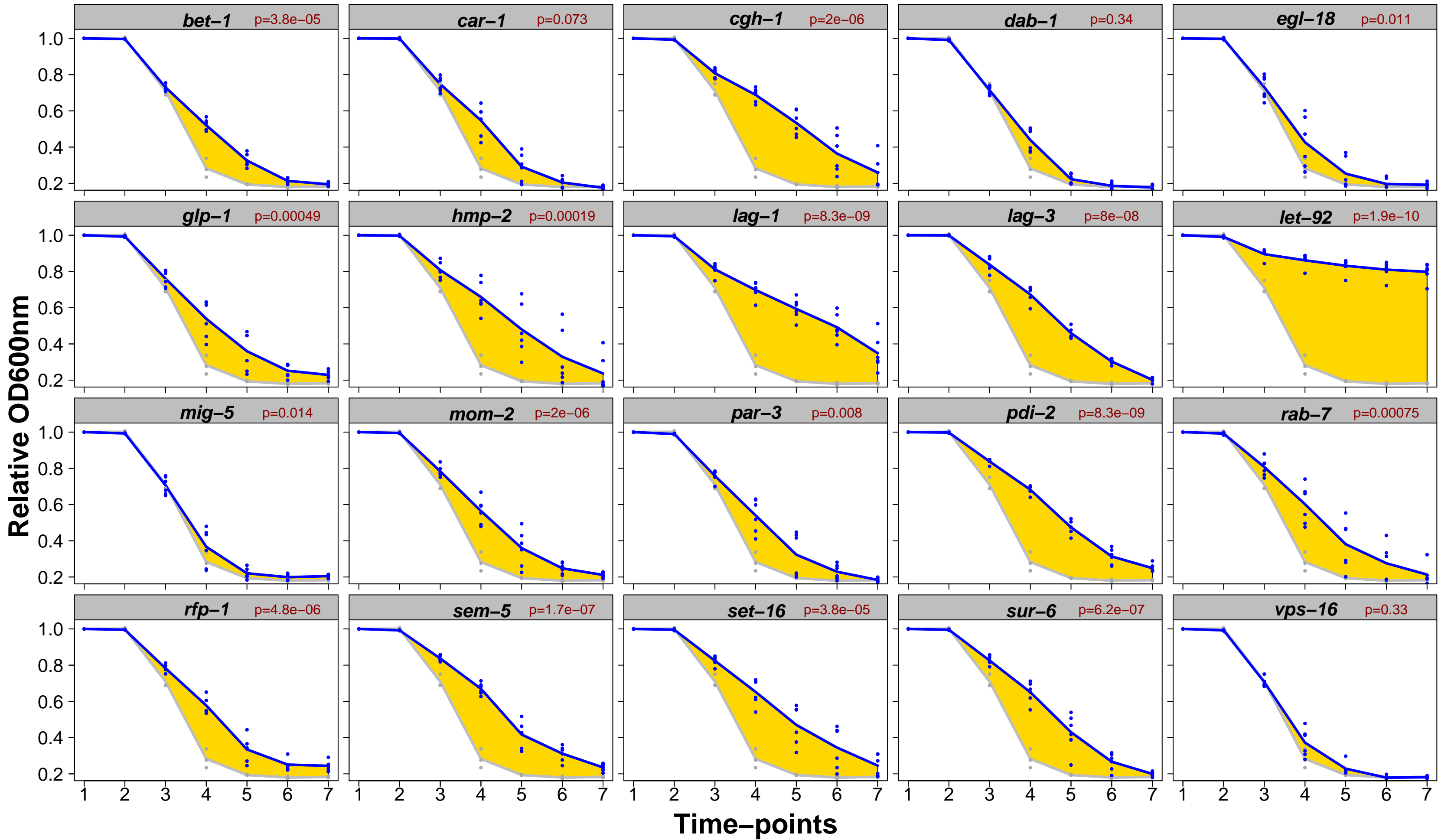

Supplement: Additional file 3 — 20 randomly selected RNAi treatments predicted to affect viability. Fitness Curves of RNAi treatments Figure S2. 20 randomly selected RNAi treatments predicted to affect viability by RNAi according to [1]. Shown are the FCs of RNAi treatments (blue line) compared to empty vector (grey line). The difference between the RNAi effect and the control empty vector (ev) is shown in yellow. The RNAi treatment on N2 is indicated in the grey box. Individual measurements are shown as dots, blue for RNAi treatments and grey for ev. [file 1471-2164-12-510-S3.PDF]

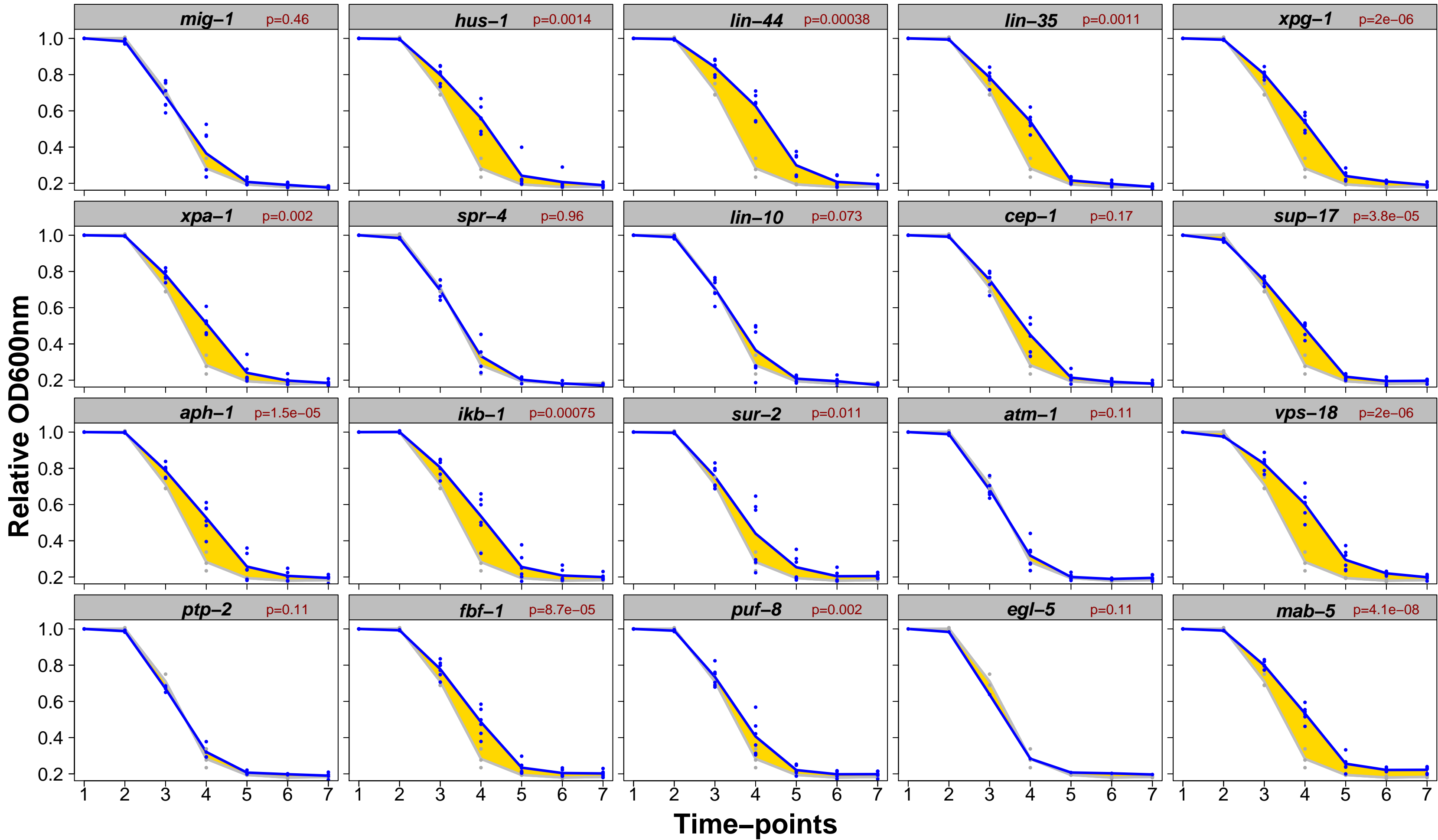

Supplement: Additional file 4 — 20 randomly selected RNAi treatments predicted to NOT affect viability. Fitness Curves of RNAi treatments Figure S3. 20 randomly selected RNAi treatments predicted to NOT affect viability by RNAi according to [1]. Shown are the FCs of RNAi treatments (blue line) compared to empty vector (grey line). The difference between the RNAi effect and the control empty vector (ev) is shown in yellow. The RNAi treatment on N2 is indicated in the grey box. Individual measurements are shown as dots, blue for RNAi treatments and grey for ev. [file 1471-2164-12-510-S4.PDF]
